# Upadacitinib-Induced Remission in Multicentric Reticulohistiocytosis: Expanding the Therapeutic Role of JAK Inhibition

**DOI:** 10.3390/ijms27062898

**Published:** 2026-03-23

**Authors:** Cristina Pamfil, Mohamed Amin Taki, Elisabeta Candrea, Laura Damian, Maia Ioana Mihon, Diana Maria Margareta Moldovan, Simona Rednic

**Affiliations:** 1 Rheumatology Department, Emergency County Clinical Hospital Cluj, 400347 Cluj-Napoca, Romania; cristinapamfil.umfcluj@gmail.com (C.P.); ldamian.reumatologie@gmail.com (L.D.); maia.mihon@gmail.com (M.I.M.); srednicumfcluj@gmail.com (S.R.); 2 Department of Dermatology, “Iuliu Hațieganu” University of Medicine and Pharmacy Cluj-Napoca, 400012 Cluj-Napoca, Romania; diana.moldovan.m@gmail.com; 3 Regional Institute of Gastroenterology and Hepatology “Octavian Fodor”, 3rd Medical Clinic, 400394 Cluj-Napoca, Romania; mohamed96.tk@gmail.com; 4 Department of Dermatology, Emergency County Clinical Hospital Cluj, 400347 Cluj-Napoca, Romania; 5 Medical and Surgical Sciences Department, University of Cordoba, 14071 Córdoba, Spain

**Keywords:** multicentric reticulohistiocytosis, histiocytosis, JAK inhibitor, upadacitinib, primary biliary cholangitis

## Abstract

Multicentric reticulohistiocytosis (MRH) is a rare systemic histiocytic disorder of uncertain etiology characterized by papulonodular cutaneous lesions and potentially destructive polyarthritis, with variable multisystem involvement. Owing to its low prevalence, evidence for optimal management remains limited, and treatment responses are heterogeneous. Emerging reports suggest that Janus kinase (JAK) inhibition may provide benefit in refractory disease. We report a 60-year-old woman with MRH presenting with papulonodular skin lesions, symmetric polyarthritis, constitutional symptoms, and interstitial lung disease (nonspecific interstitial pneumonia pattern) in the context of co-existing primary biliary cholangitis and no evidence of malignancy. Prior therapies (glucocorticoids, methotrexate, leflunomide) achieved suboptimal control. Upadacitinib, a selective JAK1 inhibitor, induced rapid and complete remission of cutaneous and articular disease with improvement of pulmonary involvement. Secondary weight gain and incident diabetes were managed with tirzepatide. This case adds to the limited literature supporting JAK inhibition as a targeted option for refractory MRH, including multisystem disease with pulmonary involvement. Systematic evaluation of efficacy, durability, and safety is warranted.

## 1. Introduction

Multicentric reticulohistiocytosis is best characterized as an immune-mediated systemic inflammatory disorder with features overlapping the autoimmune spectrum. It is defined histopathologically by dermal and synovial infiltration of activated macrophages and multinucleated giant cells with eosinophilic “ground-glass” cytoplasm, and clinically by destructive inflammatory polyarthritis and papulonodular cutaneous lesions [1,2]. Although its precise pathogenesis remains incompletely elucidated, evidence supporting an autoimmune basis includes immune system dysregulation with elevated pro-inflammatory cytokines (e.g., TNF-α, IL-1, IL-6), frequent coexistence with other autoimmune connective tissue diseases, and clinical responsiveness to immunomodulatory and biologic therapies targeting inflammatory pathways [1,2]. Collectively, these findings support the classification of MRH within the spectrum of autoimmune or immune-mediated inflammatory diseases rather than a primary neoplastic or purely reactive histiocytic process.

Clinically, MRH presents with papulonodular cutaneous lesions—often on the upper trunk, face, and hands—and a symmetric, sometimes rapidly destructive polyarthritis that frequently involves distal interphalangeal joints and large joints such as the knees [3,4]. Extra-articular disease is heterogeneous and may involve the pulmonary, gastrointestinal, and ocular systems, among others [1,5]. An association with malignancy has been described, suggesting a potential paraneoplastic mechanism; coexistence of autoimmune diseases has been observed in a subset of patients [6].

Diagnosis requires histopathologic confirmation from synovial or cutaneous biopsies demonstrating typically CD68-positive, CD1a-negative histiocytes and multinucleated giant cells on immunohistochemistry [1,7]. Owing to rarity, evidence-based treatment algorithms are lacking. Reported therapies include systemic glucocorticoids; conventional immunosuppressants (e.g., methotrexate, azathioprine, leflunomide, cyclophosphamide, chlorambucil, cyclosporine); and biologics such as TNF-α inhibitors and tocilizumab, with variable and often incomplete responses. Given the centrality of cytokine signaling in MRH pathogenesis, Janus kinase (JAK) inhibition has emerged as a plausible strategy [7]. Although only a handful of cases have been published, outcomes have been encouraging in refractory disease [8].

Here we describe a patient with multisystem MRH—including interstitial lung disease—who achieved complete clinical and radiologic remission with the selective JAK1 inhibitor upadacitinib after failure of conventional agents.

## 2. Case Report

**Figure 1:**
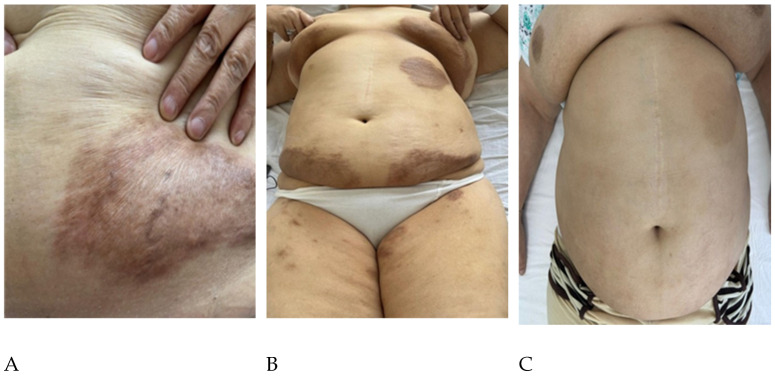
Cutaneous evolution in MRH. (**A**) Close-up of a large, brownish papulonodular plaque on the anterior chest. (**B**) Wider view showing multiple brownish patches on the thorax and proximal limbs at presentation. (**C**) Marked regression with near-complete resolution of cutaneous lesions following treatment with upadacitinib.

A 60-year-old woman presented with progressive bilateral arthralgia and stiffness of the wrists, knees, and ankles refractory to non-steroidal anti-inflammatory drugs and physiotherapy. After approximately one year, she developed non-pruritic, grey-brown- violaceous plaques on the breasts and anterior thoracic wall, some with infiltration and discrete papules in the periphery, accompanied by marked fatigue, exertional dyspnea, and unintentional weight loss (Figure 1A,B). Notably, no specific trigger was identified at onset (including recent infection, vaccination, new medication, or unusual ultraviolet/sunlight exposure).

Skin biopsy revealed a dense dermal infiltrate of histiocytes and multinucleated giant cells with eosinophilic, finely granular “ground-glass” cytoplasm, including Touton-type giant cells. The multinucleated cells were PAS-positive. Immunohistochemistry showed CD68-positive and CD1a-negative staining in histiocytes and giant cells, consistent with MRH.

The patient had grade I obesity and bilateral xanthelasma. Mild synovial swelling was present in the second to fourth metacarpophalangeal and proximal interphalangeal joints bilaterally, and in the right knee. Chest auscultation revealed bibasilar crackles and diminished vesicular breath sounds. High-resolution CT demonstrated a nonspecific interstitial pneumonia (NSIP) pattern in both lower and upper lobes. A comprehensive malignancy screen was negative. Serology showed elevated antimitochondrial antibodies and cholestatic liver enzymes, leading to a diagnosis of primary biliary cholangitis. Methotrexate (20 mg/week) produced only a short-lived improvement. Within six months, cutaneous lesions progressed, and dyspnea persisted. Switching to leflunomide conferred no benefit on skin or pulmonary symptoms and only minimal improvement in arthralgia (Figure 1B).

Given refractory multisystem disease, upadacitinib was initiated. By week 6, cutaneous lesions and joint symptoms improved markedly. At 12 months, there was complete clinical remission of cutaneous and articular manifestations, with improvement in ground-glass opacities on follow-up CT and in DLCO values (47% to 53% of predicted). Adverse events included significant weight gain (≈25 kg) and new-onset diabetes; tirzepatide was introduced with metabolic monitoring. Notably, baseline liver biochemistry was assessed before initiating treatment (DMARDs and upadacitinib) and monitored during follow-up. Transaminases and bilirubin remained within the reference range throughout treatment. A mild cholestatic pattern (slightly increased GGT and ALP, less than <x1.5 upper normal range) was present at baseline and persisted without clinically significant change. No serious infections or thromboembolic events occurred during follow-up.

## 3. Discussion

MRH remains a therapeutically challenging, systemically heterogeneous non-Langerhans cell histiocytosis in which papulonodular skin disease and aggressive, potentially mutilating polyarthritis may coexist with variable multisystem involvement [1]. Because controlled data are lacking, management has relied on glucocorticoids, conventional DMARDs, and biologics with inconsistent depth and durability of response [7]. The expanding single-case experience with JAK inhibitors is therefore clinically meaningful, but it also raises a mechanistic question that aligns with the molecular scope of IJMS: which dominant signaling programs in MRH are interrupted by JAK blockade, and why might some patients fail to respond?

At the tissue level, MRH lesions contain CD68^+^/CD163^+^ histiocytes and multinucleated giant cells with “ground-glass” cytoplasm and a non-Langerhans immunophenotype. Functional data support an osteoclast-like differentiation state (e.g., TRAP/cathepsin K expression) and implicate an osteoclastogenic microenvironment in which RANKL, M-CSF, and TNF-α promote bone resorption and structural damage [9,10]. In parallel, MRH exhibits a cytokine-driven inflammatory program, with prominent roles proposed for TNF-α, IL-1β, IL-6, IL-12, and interferon-related signaling [11]. Many of these inflammatory inputs converge on JAK–STAT, particularly through JAK1/2 and TYK2, providing a biologically plausible rationale for JAK inhibition in refractory MRH [8].

Within the JAK family, JAK1 is positioned at a convergence point for receptor systems that are directly relevant to macrophage activation and tissue inflammation. Gp130 cytokines such as IL-6 recruit JAK1 (together with JAK2/TYK2) and drive STAT3-dependent transcription; type I interferons signal through JAK1/TYK2 and interferon-γ through JAK1/JAK2, amplifying STAT1 programs; and common γ-chain cytokines (e.g., IL-2/IL-4/IL-15) engage JAK1/JAK3, supporting lymphocyte-derived inflammatory cues. By inhibiting JAK1, upadacitinib can blunt multiple upstream inputs that sustain histiocytic activation, cytokine amplification loops, and downstream chemokine networks that recruit and polarize myeloid cells [12].

This mechanism is also compatible with the osteoclast-like biology of MRH. Although RANKL-driven osteoclastogenesis is not directly blocked by JAK inhibitors, suppression of IL-6/STAT3- and interferon-linked inflammatory transcription may indirectly reduce the availability of pro-resorptive mediators and the inflammatory amplification loops that reinforce RANKL/M-CSF/TNF-α activity within synovial and periarticular tissues [9,10,12]. Importantly, JAK inhibitors are not mechanistically interchangeable: differences in JAK isoform selectivity and cytokine coverage translate into heterogeneous biologic and clinical effects across immune-mediated diseases [13]. In MRH—where both macrophage/osteoclast programs and cytokine-driven inflammation appear central—JAK1-selective inhibition is attractive because it preferentially targets IL-6 and interferon signaling while still dampening several lymphocyte- and myeloid-derived cytokine circuits that feed the lesional macrophage population [8,12].

Our patient’s course—early improvement within weeks and complete remission of cutaneous and articular disease at 12 months—fits a model in which a cytokine/JAK–STAT-dependent circuit is a dominant, druggable driver of disease activity. The parallel improvement of NSIP-type interstitial lung disease, with regression of ground-glass changes and a rise in DLCO, further supports a systemic effect on pathways (notably IL-6 and interferon signaling) that are established mediators of tissue inflammation and remodeling [12]. Although causality cannot be proven in a single case, concordant kinetics across organs is more consistent with upstream pathway interruption than with isolated symptomatic control.

At the same time, MRH is increasingly recognized as biologically heterogeneous. Genomic and transcriptomic studies have identified MAPK-pathway alterations (e.g., MAP2K1) and kinase fusions (e.g., KIF5B–FGFR1) in a subset of cases, supporting clonal/neoplastic biology alongside inflammation [14]. These observations suggest that MRH may span a spectrum from predominantly cytokine-driven inflammatory disease (more likely to be JAK inhibitor–responsive) to phenotypes in which autonomous MAPK signaling is a primary engine of histiocytic proliferation and tissue injury. In such cases, JAK inhibition may not sufficiently suppress the dominant driver, offering a biologically plausible explanation for therapeutic failures and underscoring the need for mechanism-guided escalation strategies in non-responders.

Published experience since 2021 is limited but informative: case reports describe rapid clinical remission of MRH with tofacitinib, upadacitinib, or baricitinib after inadequate response to glucocorticoids and/or conventional or biologic DMARDs (.) [15,16,17,18,19]. Conversely, at least one non-response to baricitinib has been reported [20]. Two non-mutually exclusive explanations warrant consideration: (i) inter-patient biological heterogeneity (including clonal/MAPK-driven disease) [14], and (ii) pharmacologic differences between JAK inhibitors that may matter when a specific cytokine axis is dominant [13]. Together, these observations caution against assuming a uniform “class effect” and support viewing treatment response as a mechanistic readout. A summary of published MRH cases treated with JAK inhibitors, including the present case, is provided in Table 1.

Creating space for non-responders is particularly important in a rare disease where randomized trials are unlikely. Future case series and registries should, when feasible, incorporate lesional molecular profiling (targeted NGS for MAPK pathway genes and actionable kinase fusions) alongside immunophenotyping and pathway readouts (e.g., STAT1/STAT3 activation signatures) to correlate molecular phenotypes with treatment outcomes [14]. Such data could enable a pragmatic stratification model: patients without MAPK drivers and with prominent cytokine/JAK–STAT signatures could be prioritized for JAK inhibition, whereas those harboring MAPK alterations—or those failing an initial JAK inhibitor—could be evaluated for pathway-targeted approaches (e.g., MEK or FGFR inhibition) or rational combinations that address both inflammatory amplification and clonal signaling [14,20].

Extra-articular involvement may provide additional mechanistic clues. Pulmonary disease in MRH is uncommon but increasingly recognized, spanning fibrotic and inflammatory interstitial patterns and, in rare instances, tissue infiltration by histiocytes mirroring cutaneous pathology [21]. In our patient, improvement of NSIP changes in parallel with skin and joint remission suggests that lung involvement can, at least in some cases, be part of the same systemic cytokine program. This supports systematic respiratory phenotyping and longitudinal imaging/physiology in future JAK inhibitor–treated cases, to determine whether pulmonary endpoints track with molecular and clinical response [20].

The relationship between MRH and autoimmunity also supports a shared immune dysregulation model. In early stages, symmetric polyarthritis can closely mimic rheumatoid arthritis, with the diagnosis becoming apparent only as cutaneous or systemic features evolve [21]. In the Mayo Clinic experience, approximately one-third of MRH patients had a concurrent autoimmune condition, most frequently rheumatoid arthritis [7], and associations with systemic lupus erythematosus, Sjögren’s syndrome, juvenile idiopathic arthritis, immune thrombocytopenic purpura, multiple sclerosis, and other immune-mediated phenotypes have been reported [7,22,23]. Such clustering is compatible with a macrophage-centered inflammatory network sustained by cytokines and interferon-related pathways—again aligning conceptually with JAK–STAT as a tractable node [8,13].

Our case also extends the limited evidence linking MRH with primary biliary cholangitis (PBC), previously supported by only a single published report [24]. Whether this association reflects shared predisposition (e.g., dysregulated innate immunity and tissue-specific autoimmunity) or coincidental co-morbidity remains unknown. Nevertheless, it provides a clinical rationale for comprehensive autoimmune screening in MRH and suggests that systems-level immunomodulation may be particularly relevant in patients presenting with overlapping autoimmune features.

The co-occurrence of PBC in our patient also highlights the concept of a skin-liver axis, in which bile acids, pruritogens, and cytokine signaling mediate bidirectional mechanisms between hepatobiliary disease and the skin [25,26,27]. In cholestatic disorders such as PBC, retention of bile acids and other pruritogenic mediators can activate cutaneous sensory neurons and influence keratinocyte and immune-cell responses via bile acid receptors (including TGR5), contributing to itch, excoriations, and inflammatory skin changes [25,28]. Conversely, systemic inflammation originating from skin and joint disease can modulate hepatic immune activation through circulating cytokines and the gut-liver-skin network [27]. While our patient’s MRH lesions were not pruritic, the presence of xanthelasma and cholestatic biochemistry underscores the need to interpret cutaneous findings in the context of underlying cholestasis and to monitor liver-related symptoms during immunomodulatory therapy.

Therapeutic enthusiasm must be balanced by safety considerations and long-term uncertainty. Our patient developed substantial weight gain and incident diabetes during follow-up; while causality cannot be definitively attributed in a single case, weight gain has been reported with oral JAK inhibitors in systematic analyses, and upadacitinib is known to affect lipid parameters [29]. Upadacitinib has also been associated with asymptomatic transaminase elevations; product information recommends baseline assessment and periodic monitoring, with prompt evaluation of any elevations to identify potential drug-induced liver injury (DILI), although clinically apparent DILI appears rare [30,31,32]. More broadly, JAK inhibitors require individualized risk stratification and monitoring for infections, cytopenias, thromboembolic events, and cardiovascular complications, particularly when long-term exposure is anticipated [12]. In MRH, defining the optimal duration of JAK inhibition, the feasibility of dose reduction after remission, and the relapse risk after discontinuation are key unanswered questions.

Several limitations should therefore be stated explicitly. First, single-case observations cannot establish efficacy or generalizability, and spontaneous fluctuation or delayed effects of prior therapies cannot be fully excluded. Second, molecular profiling was not performed in our patient; consequently, we cannot link response to a defined molecular subtype (e.g., cytokine-dominant versus MAPK-altered disease) [14]. Third, extra-articular improvement—including pulmonary findings—should be interpreted cautiously in the absence of histologic confirmation and standardized longitudinal endpoints. Despite these limitations, the depth and durability of remission in this multisystem case support JAK1 inhibition as a rational targeted option for refractory MRH. Practically, we propose: (i) histologic confirmation; (ii) comprehensive malignancy and autoimmune screening [7]; (iii) baseline multisystem staging (skin, joints, lung); (iv) consideration of lesional NGS when available [13]; and (v) early escalation to targeted therapy—such as JAK inhibition—when progressive disease threatens function. For patients who do not respond, integrating molecular drivers (e.g., MAPK alterations/fusions) with pathway-based treatment selection represents a clear research priority [14,19].

All published cases (7–10) reported complete remission within ~5 months of JAK inhibitor initiation; no relapses were reported during the reported follow-up.

## Figures and Tables

**Table 1:** Literature review of MRH cases treated with JAK inhibitors.

Case (Year)	Age/Sex	Comorbidities	Systemic Features (Selected)	Biopsy/Immunophenotype	Prior Therapies	JAK Inhibitor	Outcome
**Bruscas Izu (2021)** [15]	41/M	NA	Weight loss	MRH confirmed by morphology + IHC	GC, MTX, ETN, LEF	Tofacitinib	Complete remission
**Niaki (2021)** [16]	50/F	Breast and ovarian cancer (history)	NA	Histiocytic dermal infiltrate	GC, HCQ, MTX, IFX	Upadacitinib	Complete remission
**Chen (2022)** [17]	64/M	NA	NA	Multinucleated histiocytes with ground-glass cytoplasm; CD68+, CD163+, CD1a–	GC, HCQ	Baricitinib	Complete remission
**Fan (2023)** [18]	57/F	Eosinophilic gastroenteritis	NA	Not performed	GC, NSAIDs	Tofacitinib	Complete remission
**Mercader-Salvans J (2025)** [19]	42/F	Sjögren’s disease	NA		GC, HCQ, MTX, ADA	Tofacitinib	
**Present case**	60/F	Primary biliary cholangitis	Fatigue, weight loss, interstitial lung disease (NSIP)	Histiocytic infiltrate with multinucleated giant cells; PAS+; CD68+, CD1a–	NSAIDs, MTX, LEF	Upadacitinib	Complete remission (skin, joints) + CT improvement

Abbreviations: F, female; M, male; NA, not available; GC, glucocorticoids; HCQ, hydroxychloroquine; MTX, methotrexate; LEF, leflunomide; ETN, etanercept; IFX, infliximab; ADA, adalimumab; NSAIDs, non-steroidal anti-inflammatory drugs; IHC, immunohistochemistry; NSIP, nonspecific interstitial pneumonia.

## Data Availability

Data supporting this case report are contained within the article.
